# Binding of Selected Ligands to Human Protein Disulfide Isomerase and Microsomal Triglyceride Transfer Protein Complex and the Associated Conformational Changes: A Computational Molecular Modelling Study

**DOI:** 10.1002/open.202400034

**Published:** 2025-01-31

**Authors:** Yong Xiao Yang, Peng Li, Bao Ting Zhu

**Affiliations:** ^1^ Shenzhen Key Laboratory of Steroid Drug Discovery and Development School of Medicine The Chinese University of Hong Kong Shenzhen, Guangdong 518172 China; ^2^ Shenzhen Bay Laboratory Shenzhen 518055 China

**Keywords:** Human Protein Disulfide Isomerase, Microsomal Triglyceride Transfer Protein Complex, Binding of Selected Ligands, MTP Inhibition, Associated Conformational Changes

## Abstract

Human protein disulfide isomerase (PDI) is a multifunctional protein, and also serves as the β subunit of the human microsomal triglyceride transfer protein (MTP) complex, a lipid transfer machinery. Dysfunction of the MTP complex is associated with certain disease conditions such as abetalipoproteinemia and cardiovascular diseases. It is known that the functions of PDI or the MTP complex can be regulated by the binding of a small‐molecule ligand to either of these two proteins. In the present study, the conformational changes of the MTP complex upon the binding of three selected small‐molecule ligands (17β‐estradiol, lomitapide and a phospholipid) are investigated based on the available biochemical and structural information by using the protein–ligand docking method and molecular dynamics (MD) simulation. The ligand‐binding sites, the binding poses and binding strengths, the key binding site residues, and the ligand binding‐induced conformational changes in the MTP complex are analyzed based on the MD trajectories. The open‐to‐closed or closed‐to‐open transitions of PDI is found to occur in both reduced and oxidized states of PDI and also independent of the presence or absence of small‐molecule ligands. It is predicted that lomitapide and 1,2‐diacyl‐sn‐glycero‐3‐phosphocholine (a phospholipid) can bind inside the lipid‐binding pocket in the MTP complex with high affinities, whereas 17β‐estradiol interacts with the lipid‐binding pocket in addition to its binding to the interface region of the MTP complex. Additionally, lomitapide can bind to the ***b’*** domain of PDI as reported earlier for E_2_. Key residues for the ligand‐binding interactions are identified in this study. It will be of interest to further explore whether the binding of small molecules can facilitate the conformational transitions of PDI in the future. The molecular and structural insights gained from the present work are of value for understanding some of the important biological functions of PDI and the MTP complex.

## Graphical abstract

The interactions of MTP, MTPα and MTPβ (PDI) with three representative small‐molecule ligands are investigated using computational structural and chemical analysis to characterize the binding site structures, the binding strength, the key residues involved, and the conformational changes. The obtained new information offers valuable insights into the understanding of the mechanism of protein–protein and protein–ligand interactions and also for designing novel inhibitors of MTP and PDI.

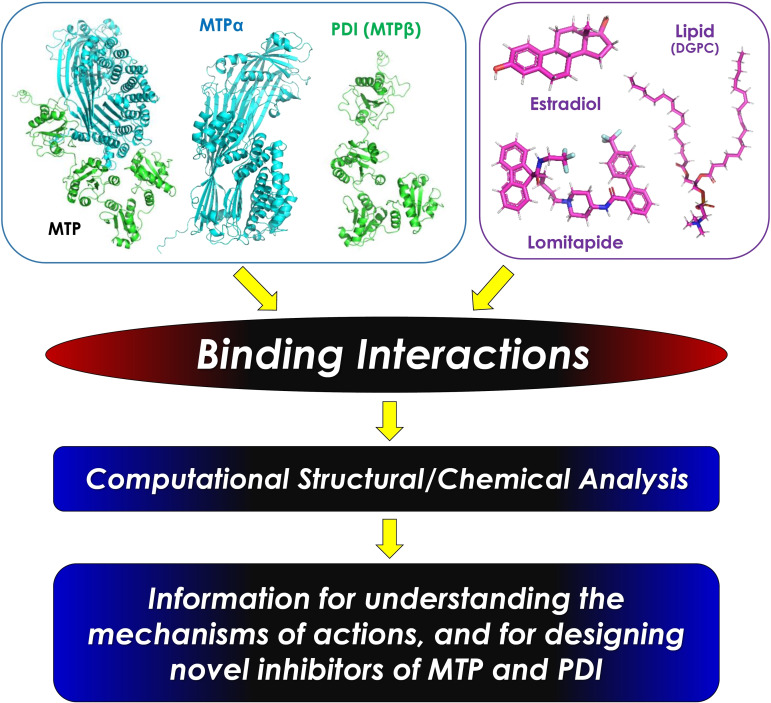

## Introduction

The human protein disulfide isomerase (PDI) is a multi‐functional protein that has many important biological functions.[^1^, ^2^, ^3^] Biochemically, the best‐known functions of PDI are related to its ability to catalyze the oxidative folding of proteins and to serve as a molecular chaperone.[^4^, ^5^] PDI also serves as the β subunit of human microsomal triglyceride transfer protein (MTP), and the other subunit of the MTP complex is its α subunit.[^6^, ^7^] The MTP complex is involved in the transfer of neutral lipids during the assembly of ApoB‐containing lipoproteins, which mostly include the very low‐density lipoproteins (VLDL) and chylomicrons.[^8^, ^9^] Dysfunction of the MTP complex and/or PDI caused by mutations or loss of functions is associated with certain disease conditions such as abetalipoproteinemia, cardiovascular diseases and neurodegeneration.[^10^, ^11^, ^12^] In addition, inhibitors of MTP or PDI have been suggested to be of potential therapeutic value for homozygous familial hypercholesterolemia.[^13^, ^14^]

Earlier experimental studies have deciphered the protein structures of PDI and MTP.[^7^, ^15^] Structurally, PDI (also commonly referred to as MTPβ subunit) has four major domains, namely, the ***a*** domain (residues 26–133), ***b*** domain (residues 137–232), ***b’*** domain (residues 235–349), and ***a’*** domain (residues 369–479).^[15]^ The main domains of the MTPα subunit in the MTP complex are the β‐barrel domain (residues 19–297), α‐helical domain (residues 298–603), and lipid‐binding domain (residues 604–894).^[7]^ The ligand‐binding sites in PDI and the MTP complex have been characterized based on their experimental structures.[^7^, ^16^, ^17^] In the present work, three representative small‐molecule ligands, *i. e*., 17β‐estradiol (E_2_), lomitapide and 1,2‐diacyl‐sn‐glycero‐3‐phosphocholine (DGPC, a phospholipid), were selected to explore the ligand‐binding interactions with PDI and the MTP complex. Earlier experimental studies have reported the biochemical properties of the binding interactions of the PDI–E_2_ complex,^[16]^ the MTP–lomitapide complex^[18]^ and the MTP–DGPC complex.[^7^, ^19^] In addition, the E_2_‐binding site in PDI has been characterized using both experimental and computational approaches.^[16]^ Our earlier study indicated that the binding of E_2_ to the MTP complex can destabilize the binding interface between PDI and MTPα subunit,^[17]^ which offers partial explanation for an estrogen receptor (ER)‐independent lipid‐modulating effect of E_2_.^[20]^ The present study sought to further explore whether there are other E_2_‐binding site(s) in the MTP complex. Lomitapide is a known MTP inhibitor which is effective for treating homozygous familial hypercholesterolemia.[^21^, ^22^] The lomitapide‐binding site in the MTP complex has been predicted in a previous work.^[23]^ However, the predicted binding site may not be as reliable since the homology model of the MTP complex in the earlier study^[23]^ differed significantly from the experimental structure.^[7]^ It is, therefore, necessary to characterize the lomitapide‐binding site based on a more‐reliable, experimentally‐determined structure of the MTP complex.^[7]^ Polyethylene glycol (PEG) was a ligand found in the lipid‐binding pocket of the experimental MTP structure.^[7]^ Since phospholipids are thought to be the native ligands for the MTP complex, a representative phospholipid (1,2‐diacyl‐sn‐glycero‐3‐phosphocholine, DGPC) was selected in this study for comparison with lomitapide and E_2_ with respect to their binding interactions with the MTP complex in terms of binding sites, binding poses and binding strengths. The conformational changes in the structures of the two proteins were analyzed based on MD trajectories. Information derived from these molecular modelling studies is useful for explaining the mechanism by which lomitapide may exert its action at the molecular/structural levels, and this information will be of value for designing new MTP inhibitors.

## Materials and Methods

### Construction of the full‐length structures of PDI and the MTP complex

Under physiological conditions, the human PDI exists in two interchangeable states, *i. e*., the reduced and oxidized states. In the known structures of human PDI available in the PDB database, there are 37 missing residues in the reduced PDI (PDB code: 4EKZ, residues 240–244, 323–324, 479–508) and 39 missing residues in the oxidized PDI (PDB code: 4EL1, chain A, residues 250–254, 320–323, 479–508), respectively.^[15]^ The missing residues were added back using SWISS‐MODEL (https://swissmodel.expasy.org/), a web server for homology modelling.^[24]^ The complete structure of the MTP complex used in this study is the structure constructed in our earlier work,^[17]^ which was modelled based on the known experimental structure (PDB code: 6I7S).^[7]^ Sequence information and experimental structures were downloaded from the Uniprot (https://www.uniprot.org/)^[25]^ and the Protein Data Bank (https://www.rcsb.org/),^[26]^ respectively. The sdf files of E_2_, lomitapide and DGPC were downloaded from PubChem (https://pubchem.ncbi.nlm.nih.gov/).[^27]^

### Prediction of the Ligand‐Binding Sites in PDI and the MTP Complex

The possible ligand‐binding sites are predicted using PrankWeb (https://prankweb.cz/), a web server for ligand‐binding site prediction based on the known protein structures.[^28^, ^29^] The protein structures used in this study included the reduced PDI (PDB code: 4EKZ), the oxidized PDI (PDB code: 4EL1, chain A), the MTP complex (PDB code: 6I7S), the reduced PDI extracted from the MTP complex (PDB code: 6I7S, chain A), and the MTPα subunit extracted from the MTP complex (PDB code: 6I7S, chain G).[^7^, ^15^]

### Construction of the structures of the PDI–ligand and MTP–ligand complexes

The structures of the protein–ligand complexes were constructed using the protein–ligand docking method. The initial ligand positions were set at the respective geometric centers of the amino acid residues for the E_2_‐binding site in PDI,^[16]^ the lipid‐binding site in the MTP complex,^[7]^ and the other ligand‐binding sites in PDI and MTP which were predicted in this study. The usefulness of the docking methodology for modeling the structure of PDI–E_2_ complex have been validated in the previous study.[^16^, ^17^] In addition, this docking method has also been used to model the binding interactions of PDI with some of the endogenous estrogen metabolites.^[30]^ At present, the experimentally‐determined structures of MTP‐lomitapide and MTP‐DGPC complexes are not available. The known lipid‐binding pocket in the experimental structure of the MTP complex^[7]^ is thus used as a reference site when lomitapide and DGPC are docked into their binding sites. After docking, the binding stabilities in the predicted structures of MTP‐ligand complexes were further compared using molecular dynamics simulation.

The complete constructed structures were processed using the Protein Preparation Wizard in Schrodinger Suite (Maestro 12.8, 2021; Schrodinger LLC, New York, NY, USA). The hydrogen atoms were added, and the protein structures were optimized using the OPLS4 force field.^[31]^ The Glide‐XP (extra precision) docking method in Schrodinger Glide software^[32]^ was employed to generate the structures of the protein–ligand complexes. The lowest energy binding poses were adopted to sample the nearby torsional minima with the Monte Carlo (MC) procedure. The centers of the docking grid box with dimensions of 30×30×30 Å^3^ was placed at the initial ligand positions in the constructed structures.

For the same protein structure and ligand, multiple binding sites were usually predicted, and multiple docking results were generated. Three scoring functions, *i. e*., X‐Score,^[33]^ PRODIGY‐LIG^[34]^ and Δ_vina_RF_20_,^[35]^ were used to further filter the docking results. Both X‐Score and PRODIGY‐LIG are linear empirical scoring functions often used for predicting the protein–ligand binding affinity.[^33^, ^34^] The former employs energy and geometric terms such as van der Waals energy, hydrogen bonding energy, deformation penalty and hydrophobic effect,^[33]^ whereas the latter uses the number of atomic contacts and electrostatic energy.^[34]^ Δ_vina_RF_20_ is a random forest‐based scoring function which relies on 20 descriptors.^[35]^ The filtered structures of the protein–ligand complexes were adopted for molecular dynamics simulation as part of additional analyses conducted in this study.

### Molecular Dynamics (MD) Simulation

To investigate the ligand‐binding stability and conformational changes in the proteins and the predicted protein–ligand complexes, molecular dynamics (MD) simulations were employed to simulate the conformational changes of PDI, MTP, the PDI–ligand complex, and the MTP–ligand complex. Molecular docking and MD simulation are the commonly‐used techniques in the computational structural biology and chemistry, and their effectiveness and usefulness are widely accepted.[^36^, ^37^]

In the protein or protein–ligand complex systems, the sidechain atoms were added using CHARMM‐GUI (https://www.charmm‐gui.org/),^[38]^ which was also used to produce the respective topology files. CHARMM General Force Field (CGenFF)^[39]^ was employed to generate the CHARMM top and par files of the ligands, and CHARMM36 m force field^[40]^ was adopted for all amino acids in proteins. All the systems were embedded into rectangular water boxes which extend the solvent 10 Å in the *x*, *y*, *z* directions. The TIP3P water model^[41]^ was adopted, and K^+^ and Cl^−^ ions were placed into the water boxes to neutralize the charge in the systems. The parameters of K^+^ and Cl^−^ ions used in this study were approximated according to Roux *et al*.^[42]^

All the systems were optimized by energy minimization with the steepest descent algorithm for 10000 steps. After energy minimization, equilibrium simulations were exerted on the systems in NVT ensemble for 2 ns with a time step of 2 fs. Langevin dynamics^[43]^ was adopted to maintain the temperature of the systems at 300 K. During the process of MD simulations, periodic boundary conditions were adopted to avoid the protein or protein–ligand complex moving out of the water box. While the particle mesh Ewald algorithm[^44^, ^45^] was employed to estimate the long‐range electrostatic interactions, the short‐range electrostatic and van der Waals interactions were truncated smoothly with a cutoff of 12 Å, and a switching function was adopted at 10 Å. Finally, the production simulations were conducted on the systems in NPT ensemble for 100 ns. The pressure of the systems was maintained at 1 atm with the Langevin piston method.^[46]^ The set of time step, the controlling of temperature, and the calculation of electrostatic and van der Waals interactions are the same as those used in the equilibrium simulation. The NAMD was used to perform the energy minimization (10000 steps), equilibrium (2 ns) and production (100 ns) simulations.^[47]^

### Metrics for Analysis of the Simulation Results

Several geometric and energy metrics were adopted to evaluate the ligand‐binding stability and characterize the conformational changes during the MD simulations. The global conformational changes of PDI were measured using one distance (**distance–0**, which is the distance between the centers of the ***a*** and ***a’*** domains) and two angles (**angle–1** is the angle between the centers of the ***a***–***b***–***b’*** domains, and **angle–2** is the angle between the centers of the ***b***–***b’***–***a’*** domains). The mean square fluctuations (MSFs) of **distance–0**, **angle–1** and **angle–2** were used to estimate the degree of the global conformational changes. The number of interface atomic contact pairs was employed to assess, from a geometric perspective, the stability of the protein–protein and protein–ligand complexes. The atomic contact pairs are the pairs with distances ≤5 Å. The predicted binding affinities of the snapshots along the MD trajectories based on X‐Score,^[33]^ PRODIGY‐LIG^[34]^ and Δ_vina_RF_20_ ^[35]^ were used to evaluate the binding stability in the protein–ligand complexes from an energy perspective.

## Resulsts and Discussion

### Structures of the PDI–ligand and MTP–ligand Complexes

**Figure 1:**
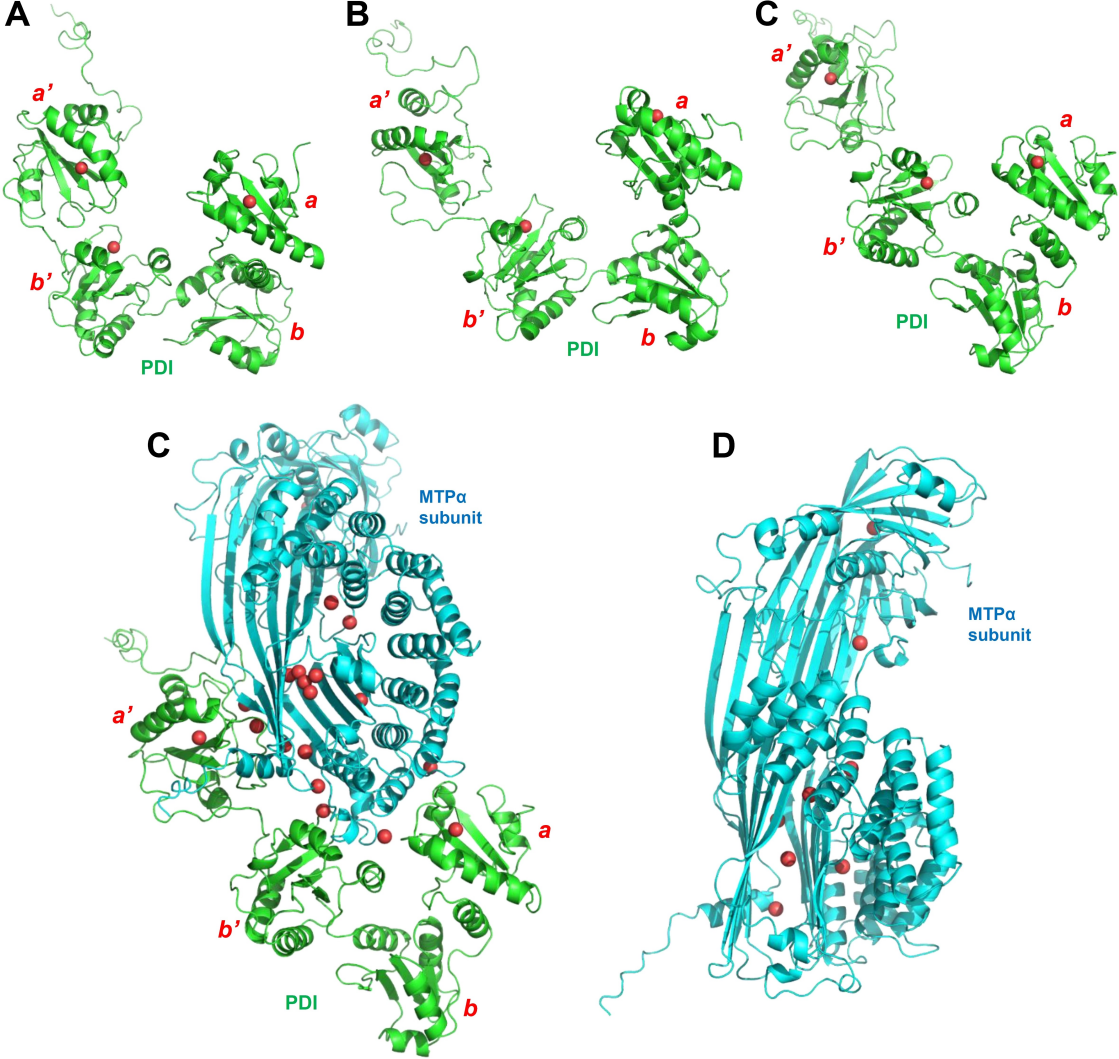
**The centers of the predicted binding pockets in the original structures using PrankWeb**.[^28^, ^29^] **A**. The centers of 3 predicted binding pockets in the reduced PDI (PDB code: **4EKZ**).^[15]^ **B**. The centers of 3 predicted binding pockets in the oxidized PDI (PDB code: **4EL1, chain A**).^[15]^ **C**. The centers of 3 predicted binding pockets in the reduced PDI taken from the MTP complex (PDB code: **6I7S, chain A**).^[7]^ **D**. The centers of 17 predicted binding pockets and the positions of the two lipid mimics as well as the center position between them in the MTP complex (PDB code: **6I7S**).^[7]^ **E**. The centers of 8 predicted binding pockets in MTPα subunit taken from the MTP complex (PDB code: **6I7S, chain G**).^[7]^ The PDI, MTPα subunit and the centers of binding pockets are colored in green, cyan and red, respectively.

Based on the known structures of PDI and the MTP complex, it was predicted by PrankWeb that there are 3 potential binding pockets for PDI, 8 potential binding pockets for MTPα subunit, and 17 potential binding pockets for the MTP complex.[^28^, ^29^] The residues surrounding the predicted potential binding pockets are listed in Supplementary Table S1. The geometric centers of the binding pockets are shown in Figure 1. While the binding pockets of PDI are located in the ***a***, ***b’*** and ***a’*** domains (Figure 1A–C), the binding pockets of the MTP complex are mostly associated with MTPα subunit and the interface regions between PDI and MTPα subunit (Figure 1D and E). Here it is of note that there is a PEG molecule (a structural mimic of lipids) found in each copy of the two experimental MTP structures (PDB code: 6I7S, chain AG or chain BH).^[7]^ After superimposing the two structures, it was found that the PEG molecule in these structures did not overlay with each other. Therefore, the potential binding sites for this lipid‐like molecule and the central position between these two binding sites are also used as the centers of the binding pockets during docking analysis.

The structures of the protein–ligand complexes were predicted using Glide‐XP (extra precision) in Schrodinger Glide.^[32]^ After the docked structures were filtered using the GlideScore,^[48]^ there were 37 predicted structures for the protein–E_2_ complexes, 73 predicted structures for the protein–lomitapide structures, and 37 predicted structures for protein–DGPC complexes. To further filter the structures, X‐Score,^[33]^ PRODIGY‐LIG^[34]^ and Δ_vina_RF_20_ ^[35]^ were used to predict the binding affinities in all 147 predicted structures of the protein–ligand complexes. For the same protein receptor and same ligand, all predicted structures of the protein‐ligand complexes are pooled and ranked according to their predicted binding energy (*k*cal/mol) and binding affinity (−log(*K*) values; here, *K* is the equilibrium dissociation constant (or inhibition constant). The predicted binding energy and binding affinity values are listed in Supplementary Tables S2–S4. Next, according to the ranking based on the binding energies/affinities from three methods, some representative structures/conformations are selected for further binding stability analysis. Specifically, the criteria for selecting these structures are as follows: ***1***) at least one rank=1 based on any one of the three affinity predictive methods; or ***2***) at least two ranks≤3 based on any two of the three methods when the number of predicted structures is ≥3 are selected initially. In addition, if the RMSD (root mean square deviation) between the structures of the protein–ligand complexes with the same protein and ligand (lomitapide or DGPC) is smaller than 3 Å, only one is retained to represent the initial structures. After further filtering, there are 37 predicted structures of the protein–ligand complexes remained.

In this study, the structure of the MTP–E_2_ complex which was reported in an earlier study^[17]^ is also used for further binding stability analysis. That structure was generated using MD simulation (50 ns) without hydrogen bond constraint.^[17]^ In addition, the structures of PDI–E_2_ and MTPα–E_2_ complexes extracted from the existing structures are also employed for further MD simulation and analysis. In total, there are 5 protein structures without ligands (Supplementary Figure S1) and 40 structures of the protein–ligand complexes which include 14 protein–E_2_ complexes (Supplementary Figure S2), 13 protein–lomitapide complexes (Supplementary Figure S3), and 13 protein–DGPC complexes (Supplementary Figure S4). For convenience of naming and denotation, the 45 systems were numbered and named (Table 1 and Supplementary Table S5). A name is composed of three parts: the system number, the protein's PDB information (including chain ID), and the ligand name; these three parts are linked together with “_”. For example, for the name 1_**6I7S‐AG**_E_2_, 1 is the system number, **6I7S‐AG** is the protein's PDB information (PDB code is **617S** and chain ID is **AG**, linked together with “‐”), and E_2_ is the ligand.

**Table 1:** Naming of the 45 protein or protein‐ligand systems.

No.	Protein (PDB code and chain ID)	Ligand	System Name	No.	Protein (PDB code and chain ID)	Ligand	System Name
1	**6I7S‐AG**	−	1_**6I7S‐AG**	24	4EL1‐A	lomitapide	24_**4EL1‐A**_Lomitapide
2	**MTP** (complex name)	E_2_	2_**MTP**_E_2_	25	4EL1‐A	DGPC	25_**4EL1‐A**_DGPC
3	**6I7S‐AG**	E_2_	3_**6I7S‐AG**_E_2_	26	4EL1‐A	DGPC	26_**4EL1‐A**_DGPC
4	**6I7S‐AG**	E_2_	4_**6I7S‐AG**_E_2_	27	6I7S‐A	−	27_**6I7S**‐A
5	**6I7S‐AG**	Lomitapide	5_**6I7S‐AG**_Lomitapide	28	PDI (subunit name)	E_2_	28_**PDI**_E_2_
6	**6I7S‐AG**	Lomitapide	6_**6I7S‐AG**_Lomitapide	29	6I7S‐A	E_2_	29_**6I7S‐A**_E_2_
7	**6I7S‐AG**	DGPC	7_**6I7S‐AG**_DGPC	30	6I7S‐A	E_2_	30_**6I7S‐A**_E_2_
8	**6I7S‐AG**	DGPC	8_**6I7S‐AG**_DGPC	31	6I7S‐A	lomitapide	31_**6I7S‐A**_Lomitapide
9	**6I7S‐AG**	DGPC	9_**6I7S‐AG**_DGPC	32	6I7S‐A	lomitapide	32_**6I7S‐A**_Lomitapide
10	**6I7S‐AG**	DGPC	10_**6I7S‐AG**_DGPC	33	6I7S‐A	lomitapide	33_**6I7S‐A**_Lomitapide
11	**4EKZ**	−	11_**4EKZ**	34	6I7S‐A	DGPC	34_**6I7S‐A**_DGPC
12	**4EKZ**	E_2_	12_**4EKZ**_E_2_	35	6I7S‐G	−	35_**6I7S**‐G
13	**4EKZ**	E_2_	13_**4EKZ**_E_2_	36	MTPα (subunit name)	E_2_	36_**MTPα**_E_2_
14	**4EKZ**	Lomitapide	14_**4EKZ**_Lomitapide	37	6I7S‐G	E_2_	37_**6I7S‐G**_E_2_
15	**4EKZ**	Lomitapide	15_**4EKZ**_Lomitapide	38	6I7S‐G	E_2_	38_**6I7S‐G**_E_2_
16	**4EKZ**	Lomitapide	16_**4EKZ**_Lomitapide	39	6I7S‐G	E_2_	39_**6I7S‐G**_E_2_
17	**4EKZ**	DGPC	17_**4EKZ**_DGPC	40	6I7S‐G	lomitapide	40_**6I7S‐G**_Lomitapide
18	**4EKZ**	DGPC	18_**4EKZ**_DGPC	41	6I7S‐G	lomitapide	41_**6I7S‐G**_Lomitapide
19	**4EL1‐A**	−	19_**4EL1‐A**	42	6I7S‐G	DGPC	42_**6I7S‐G**_DGPC
20	**4EL1‐A**	E_2_	20_**4EL1‐A**_E_2_	43	6I7S‐G	DGPC	43_**6I7S‐G**_DGPC
21	**4EL1‐A**	E_2_	21_**4EL1‐A**_E_2_	44	6I7S‐G	DGPC	44_**6I7S‐G**_DGPC
22	**4EL1‐A**	Lomitapide	22_**4EL1‐A**_Lomitapide	45	6I7S‐G	DGPC	45_**6I7S‐G**_DGPC
23	**4EL1‐A**	Lomitapide	23_**4EL1‐A**_Lomitapide				

**Figure 2:**
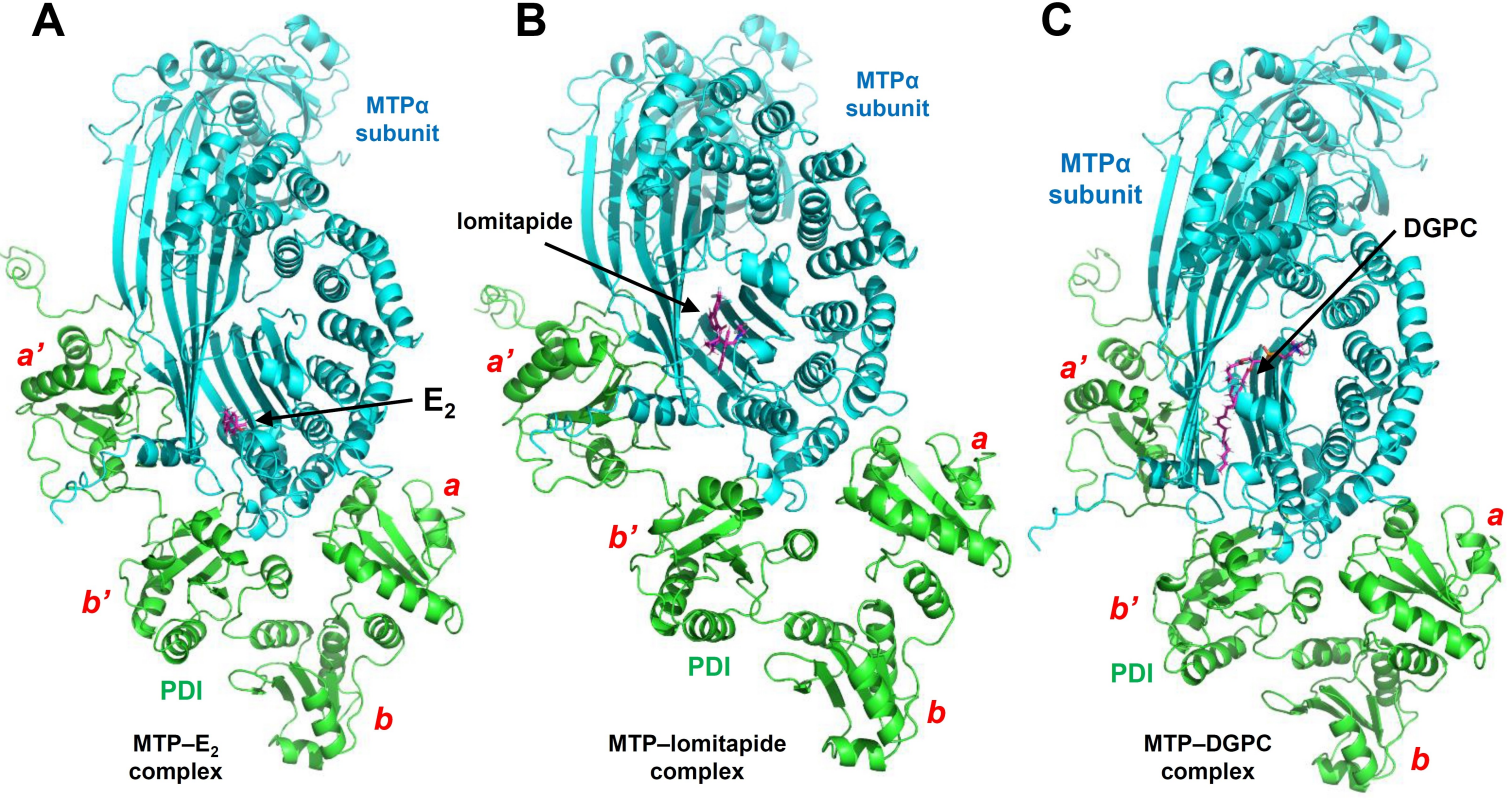
**Three examples of the structures of the MTP–ligand complexes. A**. The MTP–E_2_ complex (3_**6I7S‐AG**_E_2_). **B**. The MTP–lomitapide complex (6_**6I7S‐AG**_Lomitapide). **C**. The MTP–DGPC complex (9_**6I7S‐AG**_DGPC). The PDI, MTPα subunit and the ligands (E_2_, lomitapide and DGPC) are colored in green, cyan and purple, respectively.

The selected structures from docking calculations show that the binding sites of E_2_, lomitapide and DGPC in PDI are located in its ***b’*** domain (or the nearby region) and their binding sites in MTPα subunit are located in the lipid‐binding pocket (or the nearby region). Some ligands have been confirmed to bind with the ***b*** **’** domain of PDI using experimental or computational methods in the previous works.[^3^, ^16^, ^49^, ^50^, ^51^, ^52^] In addition, E_2_ can also bind to the interface regions between PDI and MTPα subunit (Supplementary Figure S2C). The lipid‐binding pocket has been shown in the crystal structure of MTP structure determined by Biterova et al.^[7]^ The selected examples of the predicted structures of the MTP–ligand complexes are shown in Figure 2, which include the MTP–E_2_ complex (Figure 2A), the MTP–lomitapide complex (Figure 2B), and the MTP–DGPC complex (Figure 2C). Notably, each of these three small‐molecule ligands can bind inside the lipid‐binding pocket in the MTP complex. It is the first time to discover that E_2_ can bind inside the lipid binding pocket of MTP. It is also the first time to demonstrate that lomitipde (a post‐market chemical drug targeting MTP complex) can bind inside the lipid‐binding pocket of MTP. E_2_ and lomitpide can compete with the natural substrates (such as DGPC) on their binding to MTP. The results offer a good explanation for the E_2_’s known lipid‐modulating effect, and also shed lights on the mechanism of action for the lipid‐lowering drug lomitapide from a structural chemistry perspective.

### Analysis of MD Simulation Results

The ligand‐binding stabilities and the associated conformations of PDI and MTP are explored using MD simulations in this study. Since the conformational changes during the equilibrium simulation process (2 ns) are found to be very small, the analysis is then mainly based on the production simulation process (100 ns). Compared with the longer‐time MD simulations of the PDI–rutin complex in the previous work,^[49]^ only 100 ns MD simulations are adopted in this study for the PDI–ligand complex or the MTP–ligand complex. This shorter‐time MD simulation approach is employed to compare the stabilities of the different binding positions and poses between the same ligand and protein based on the analysis of MD trajectories. Notably, similar shorter‐time MD simulations (50–100 ns) have been quite successfully used in an earlier study to discriminate the native and non‐native protein–protein docking models.^[53]^ In addition, multiple MD simulations for the same PDI–ligand or MTP–ligand complex (with different binding positions and poses) are also performed in this study (1001 representative conformations are extracted from each system based on the MD trajectories of the production simulations). This additional effort is designed to alleviate the potential limitations of the shorter‐time MD simulations.

**Characterization of ligand‐binding stability from an energy perspective**. The binding stabilities of the three selected small‐molecule ligands (E_2_, lomitapide and DGPC) with PDI or MTP are estimated using the binding energies predicted by X‐Score,^[33]^ PRODIGY‐LIG^[34]^ and Δ_vina_RF_20_.^[35]^ The binding energies are calculated based on the representative conformations of the PDI–ligand and MTP–ligand complexes selected from the production simulations. The predicted energies using X‐Score^[33]^ and PRODIGY‐LIG^[34]^ are negative, but nearly all the predicted values using Δ_vina_RF_20_ ^[35]^ are positive. When the positive values given by Δ_vina_RF_20_ ^[35]^ are higher, the corresponding structures are more stable. To coincide with the predicted energies using X‐Score^[33]^ and PRODIGY‐LIG,^[34]^ the opposite of the predicted values by Δ_vina_RF_20_ ^[35]^ was employed to represent its relative binding energies.^[35]^ The predicted binding energies of all representative conformations for all protein–ligand complexes are evenly divided into 5 bins (bin‐1 to bin‐5) based on the maximum and minimum values for each method. The conformations are categorized into the following four classes: the low binding energy confirmations, the medium binding energy confirmations, the high binding energy confirmations, and other conformations. The low‐binding‐energy conformations are those with at least two types of the binding energies belonging to bin‐1 or bin‐2; the high binding energy conformations are those with at least two types of the binding energies belonging to bin‐4 or bin‐5; the medium binding energy conformations are those with three types of binding energies not belonging to the low binding energy and high binding energy conformations; the other conformations are those without at least two types of the binding energies because of the dissociation of the protein and its ligand. The percentages of the three classes of representative conformations for each system were calculated to compare the stabilities of different protein‐ligand systems.

**Figure 3:**
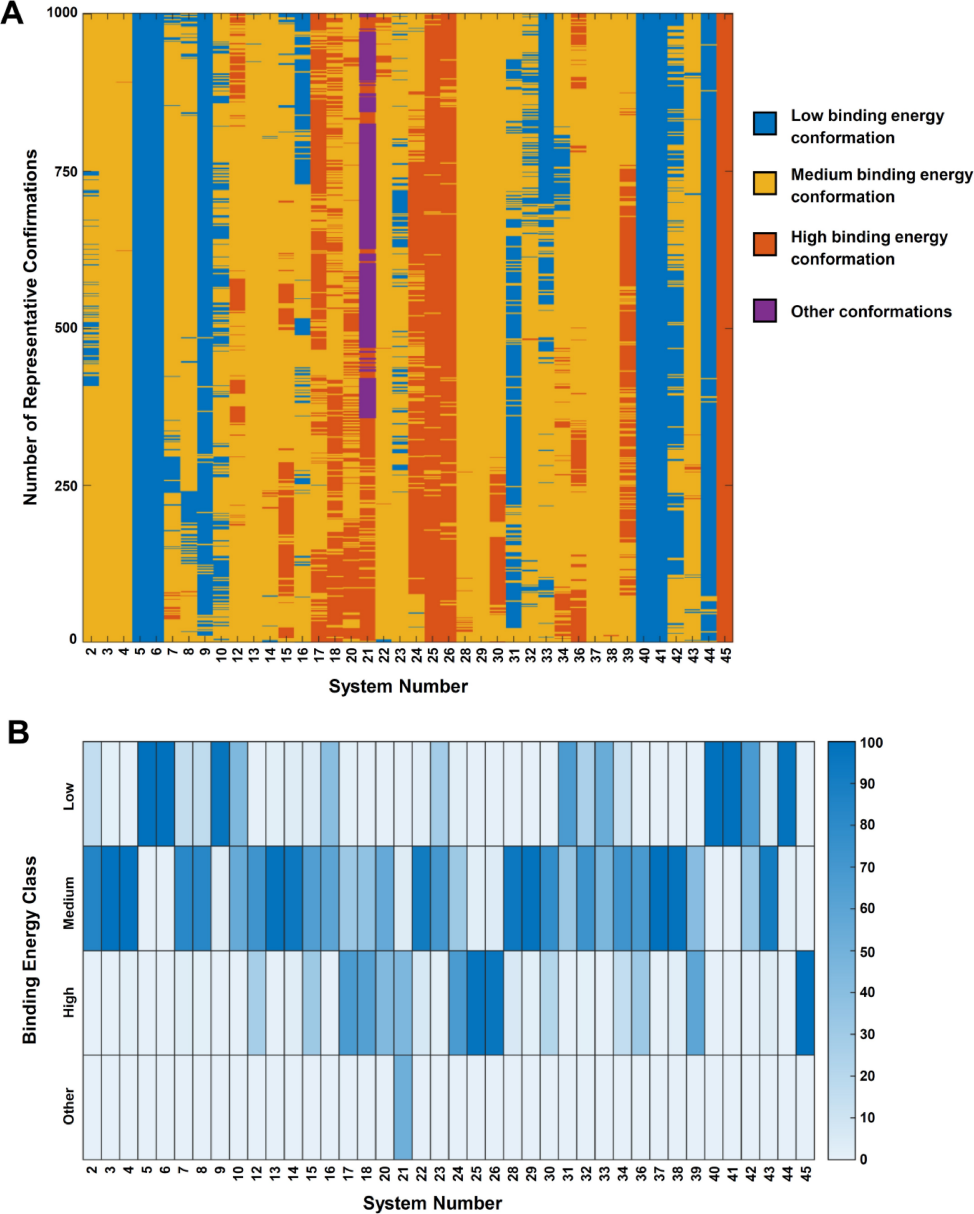
**Enegy classes of the respresentative conformations of protein‐ligand complexes during the production simulation processes (100 ns) and the percentages of the conformations belonging to different energy classes. A**. Energy classes of the represententative conformations of the 40 ligand‐containing systems. **B**. Percentages of conformations belonging to different energy classes for the 40 ligand‐containing systems (%).

The protein–ligand binding energy classes of the representative conformations of the 40 systems are shown in Figure 3A. The percentages of the low‐binding‐energy, medium‐binding‐energy and high‐binding‐energy conformations are in Figure 3B (the data are stored in Supplementary Table S6). There exist several protein–ligand systems for which most of the conformations belong to one of the following three energy classes throughout the whole simulation process, *i. e*., the low binding energy class, the high binding energy class, or the medium binding energy class.

According to the percentages of different classes of conformations, the protein–ligand systems are categorized into 7 levels (Table 2) which are employed to reflect the overall binding stabilities. For a given protein–ligand system, more conformations belong to low‐binding‐energy class, the protein–ligand binding is more stable. The systems belonging to level 1 to 4 (with high percentages of low binding energy or medium binding energy conformations and low percentages of high binding energy conformations) have relative stabilities and are possible to exist in the real biochemical environments. Those belonging to level 5 to 7 (with high percentages of high binding energy conformations) are not stable in terms of the protein–ligand binding sites and poses.

**Table 2:** Levels of different protein‐ligand systems based on the predicted binding energies.

Level	Name of system	Number of systems
1	5_**6I7S‐AG**_Lomitapide, 6_**6I7S‐AG**_Lomitapide, 40_**6I7S‐G**_Lomitapide, 41_**6I7S‐G**_Lomitapide, 44_**6I7S‐G**_DGPC, 9_**6I7S‐AG**_DGPC	6
2	42_**6I7S‐G**_DGPC, 31_**6I7S‐A**_Lomitapide, 33_**6I7S‐A**_Lomitapide, 10_**6I7S‐AG**_DGPC	4
3	16_**4EKZ**_Lomitapide, 23_**4EL1‐A**_Lomitapide, 32_**6I7S‐A**_Lomitapide, 8_**6I7S‐AG**_DGPC, 2_**MTP**_E_2_	5
4	7_**6I7S‐AG**_DGPC, 43_**6I7S‐G**_DGPC, 14_**4EKZ**_Lomitapide, 22_**4EL1‐A**_Lomitapide, 13_**4EKZ**_E_2_, 3_**6I7S‐AG**_E_2_, 37_**6I7S‐G**_E_2_, 4_**6I7S‐AG**_E_2_, 29_**6I7S‐A**_E_2_, 38_**6I7S‐G**_E_2_, 28_**PDI**_E_2_	11
5	34_**6I7S‐A**_DGPC, 30_**6I7S‐A**_E_2_, 12_**4EKZ**_E_2_	3
6	36_**MTPα**_E_2_, 15_**4EKZ**_Lomitapide, 20_**4EL1‐A**_E_2_, 39_**6I7S‐G**_E_2_, 18_**4EKZ**_DGPC, 17_**4EKZ**_DGPC, 24_**4EL1‐A**_Lomitapide, 21_**4EL1‐A**_E_2_	8
7	26_**4EL1‐A**_DGPC, 25_**4EL1‐A**_DGPC, 45_**6I7S‐G**_DGPC	3

**Notes: Level 1**: low binding energy conformations >95 %. **Level 2**: low binding energy conformations >40 %; medium binding energy conformations >50 %. **Level 3**: low binding energy conformations <15 %; medium binding energy conformations >60 %. **Level 4**: low binding energy **conformations** <15 %; % medium binding energy conformations >80 %. **Level 5**: medium binding energy **conformations** >70 %; high binding energy conformations >15 %. **Level 6**: medium binding energy **conformations** <70 %; high binding energy conformations >30 %. **Level 7**: high binding energy conformations >95 %.

Overall, lomitapide and DGPC can bind in the lipid‐binding site in the MTP complex or MTPα subunit with high binding stabilities (level 1 and 2: 5_**6I7S‐AG**_Lomitapide, 6_**6I7S‐AG**_Lomitapide, 40_**6I7S‐G**_Lomitapide, 41_**6I7S‐G**_Lomitapide, 44_**6I7S‐G**_DGPC, 9_**6I7S‐AG**_DGPC, 42_**6I7S‐G**_DGPC, 10_**6I7S‐AG**_DGPC). Similarly, lomitapide can bind to the ***b’*** domain in reduced PDI (level 2 and 3: 31_**6I7S‐A**_Lomitapide, 33_**6I7S‐A**_Lomitapide, 16_**4EKZ**_Lomitapide, 32_**6I7S‐A**_Lomitapide), and it can also bind to the *x*‐linker between the ***b’*** and ***a’*** domains in oxidized PDI (level 3: 23_**4EL1**‐A_Lomitapide).

E_2_ can bind to the interfaces between MTPα subunit and PDI (level 3 and 4: 2_**MTP**_E_2_, 4_**6I7S‐AG**_E_2_) and in the lipid‐binding pocket in MTP complex and MTPα subunit (level 4: 3_**6I7S‐AG**_E_2_, 37_**6I7S‐G**_E_2_, 38_**6I7S‐G**_E_2_). E_2_ can also bind to the ***b’*** domain in reduced PDI (level 4: 13_**4EKZ**_E_2_, 29_**6I7S‐A**_E_2_, 28_**PDI**_E_2_). The binding strength between E_2_ and oxidized PDI (level 6: 20_**4EL1‐A**_E_2_, 21_**4EL1‐A**_E_2_) is not as strong as its binding to the reduced PDI. DGPC may not bind to PDI (level 5, 6 and 7: 34_**6I7S‐A**_DGPC, 18_**4EKZ**_DGPC, 17_**4EKZ**_DGPC, 26_**4EL1‐A**_DGPC, 25_**4EL1‐A**_DGPC). E_2_ and DGPC likely also do not bind to the interacting site of MTPα subunit with PDI's ***b’*** domain and the nearby lipid‐binding pocket in MTPα subunit (level 6 and 7: 36_**MTPα**_E_2_, 39_**6I7S‐G**_E_2_, 45_**6I7S‐G**_DGPC).

**Identification of key residues for ligand binding from a geometric perspective**. The key residues involved in ligand binding interactions were derived from some of the representative systems with relative protein–ligand binding stabilities. There are 19 representative systems which are divided into 8 groups with different receptors, ligands and binding sites: MTP–lomitapide (lipid‐binding pocket), MTP–DGPC (lipid‐binding pocket), reduced PDI–lomitapide (***b’*** domain), MTP–E_2__1 (***b’*** domain, MTPα–PDI interface), oxidized PDI–lomitapide (*x*‐linker between ***b’*** and ***a’*** domains), MTP–E_2__2 (lipid‐binding pocket), MTP–E_2__3 (between ***a*** and ***b’*** domains, MTPα–PDI interface), reduced PDI–E_2_ (***b’*** domain). Here, the key ligand‐binding residues are those with the highest numbers of atomic contacts with ligand during the production simulations (100 ns). The results on distance‐based atomic contacts may not fully coincide with the results from the energy decomposition perspective. The geometrically important residues in the representative systems are also of great value for understanding the protein–ligand binding and also for the design of new drugs that can interrupt the protein–ligand binding. The residue–ligand atomic contacts are counted in the representative conformations of the representative systems. The top 10 residues with the highest numbers of ligand atomic contacts are shown in Table 3. The number of key residues involved in the binding interaction is related to the sizes of the ligand and the ligand‐binding site. For instance, in groups 4 and 7 (MTP–E_2__1 (***b’*** domain, MTPα‐PDI interface) and MTP–E_2__3 (between ***a*** and ***b’*** domains, MTPα–PDI interface)), the number of key residues of PDI (chain A) involved in binding interaction is higher than the number of key residues of MTPα subunit (chain G), indicating that PDI may play a more important role in E_2_ binding to the interface region between PDI and MTPα subunit. The high number of key residues involved in E_2_ binding interaction inside the lipid‐binding pocket (group 6) may be due to the small size of E_2_ and the large space of the lipid‐binding pocket.

**Table 3:** **Table** **3** Key ligand binding residues in the representative systems.

Group No.	Name of group	Name of system	Level	Binding site	Key ligand binding residues
1	MTP–lomitapide	5_**6I7S‐AG**_Lomitapide 6_**6I7S‐AG**_Lomitapide 40_**6I7S‐G**_Lomitapide 41_**6I7S‐G**_Lomitapide	1	lipid binding pocket	G: 635, 642, **643**, 646, 648, **666**, **671**, **706**, **707**, 716, **717**, 725, 727, **765**, **767**, **776**, 778, 815, 817, 828
2	MTP–DGPC	44_**6I7S‐G**_DGPC 9_**6I7S‐AG**_DGPC	1	lipid binding pocket	G: **643**, 664, **666**, **671**, 674, 692, **706**, **707**, 716, **717**, 725, **765**, **767**, **776**, 778, 813
3	reduced PDI–lomitapide	31_**6I7S‐A**_Lomitapide 33_**6I7S‐A**_Lomitapide 16_**4EKZ**_Lomitapide 32_**6I7S‐A**_Lomitapide	2, 3	***b’*** domain	A: 52, 97, 98, 99, **240**, **249**, 250, 254, **256**, **258**, 300, **301**, **304**, 305, **318**, 320, 324
4	MTP–E_2__1	2_**MTP**_E_2_	3	***b’*** domain, MTPα–PDI interface	A: **240**, 248, **249**, **256**, **258**, **301**, **304**, **318** G: 603, 605
5	oxidized PDI–lomitapide	23_**4EL1‐A**_Lomitapide	3	*x*‐linker between ***b’*** and ***a’*** domains	A: 304, 356, 358, 427, 428, 429, 430, 431, 437, 440
6	MTP–E_2__2	3_**6I7S‐AG**_E_2_ 37_**6I7S‐G**_E_2_ 38_**6I7S‐G**_E_2_	4	lipid binding pocket	G: 242, 642, **643**, 650, 652, 654, 661, 662, **666**, **671**, 674, 675, 697, **706**, **707**, 713, 714, **717**, 719, 757, **765**, **767**, 769, 774, **776**, 782, 784
7	MTP–E_2__3	4_**6I7S‐AG**_E_2_	4	between ***a*** and ***b’*** domains, MTPα–PDI interface	A: 97, 98, 99, 245, 246, 249, 250 G: 601, 604, 607
8	reduced PDI–E_2_	13_**4EKZ**_E_2_ 29_**6I7S‐A**_E_2_ 28_**PDI**_E_2_	4	***b’*** domain	A: **240**, 245, 248, **249**, **256**, **258**, 300, **301**, **304**, 305, **318**, 320, 324, 396, 438

According to the results of groups 1, 2 and 6, *i. e*., MTP–lomitapide (lipid‐binding pocket), MTP–DGPC (lipid‐binding pocket) and MTP–E_2__2 (lipid‐binding pocket), respectively, the common key residues in the lipid‐binding pocket involved in the binding interaction with lomitapide, DGPC and E_2_‐binding include residues 643, 666, 671, 706, 707, 717, 765, 767 and 776. The existences of these common key residues suggest a competitive relationship in the binding interaction of lomitapide, DGPC and E_2_ with the lipid‐binding site in the MTP complex. Similarly, according to the results of groups 3, 4 and 8 which include reduced PDI–lomitapide (***b’*** domain), MTP–E_2__1 (***b’*** domain, MTPα–PDI interface) and reduced PDI–E_2_ (***b’*** domain), the common key residues of the ***b’*** domain involved in the binding interaction with lomitapide and E_2_ are residues 240, 249, 256, 258, 301, 304 and 318. The existences of these common key residues suggest a competitive relationship in the binding interaction of lomitapide and E_2_ with DPI's ***b*** **’** domain.

**Figure 4:**
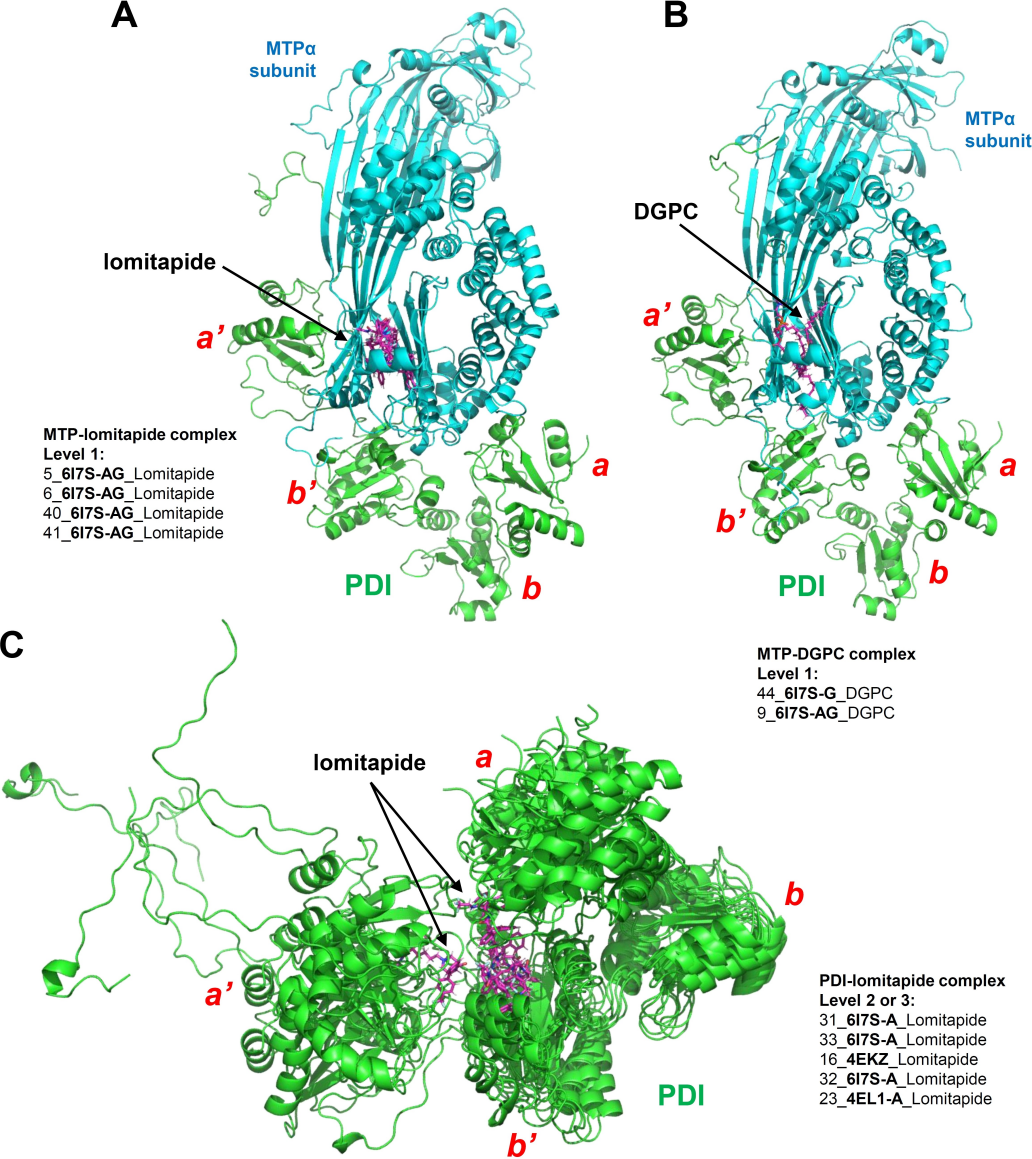
**The conformations of MTP–lomitipade, MTP–DGPC, PDI–lomitapide complexes after production simulation (100 ns). A**. MTP–lomitapide complexes (Level 1: 5_**6I7S‐AG**_Lomitapide, 6_**6I7S‐AG**_Lomitapide, 40_**6I7S‐AG**_Lomitapide, 41_**6I7S‐AG**_Lomitapide). **B**. MTP–DGPC complexes (Level 1: 44_**6I7S‐G**_DGPC, 9_**6I7S‐AG**_DGPC). **C**. PDI–lomitapide complexes (Level 2 or 3: 31_**6I7S‐A**_Lomitapide, 33_**6I7S‐A**_Lomitapide, 16_**4EKZ**_Lomitapide, 32_**6I7S‐A**_Lomitapide, 23_**4EL1‐A**_Lomitapide).

**Mechanism for MTP inhibition by lomitapide from both structure and energy perspectives**. The binding strengths in MTP–lomitapide complexes (Figure 4A, level 1: 5_**6I7S‐AG**_Lomitapide, 6_**6I7S‐AG**_Lomitapide, 40_**6I7S‐G**_Lomitapide, 41_**6I7S‐G**_Lomitapide) are comparable to the binding strengths of a phospholipid molecule (DGPC) in the MTP complex (Figure 4B, level 1: 44_**6I7S‐G**_DGPC, 9_**6I7S‐AG**_DGPC). There are 12 common key binding residues for the MTP–lomitapide and MTP–DGPC interactions (residues 643, 666, 671, 706, 707, 716, 717, 725, 765, 767, 776 and 778; Table 3). The results indicate that lomitapide has sufficient competitive ability to bind inside the MTP's lipid‐binding pocket even when a lipid ligand (such as DGPC) is present. Additionally, lomitapide can also bind to the ***b’*** domain or the *x*‐linker between the ***b’*** and ***a’*** domains of PDI (Figure 4C, level 2 or 3: 31_**6I7S‐A**_Lomitapide, 33_**6I7S‐A**_Lomitapide, 16_**4EKZ**_Lomitapide, 32_**6I7S‐A**_Lomitapide, 23_**4EL1‐A**_Lomitapide). The binding of lomitapide to PDI can also affect the PDI–MTPα interactions and the formation and stability of the MTP complex. Therefore, it is expected that the behaviors of lomitapide will have an overall negative effect on the MTP's lipid‐transfer activity.

In summary, since lomitapide can bind to MTP's lipid‐binding pocket with high affinity (comparable to MTP's natural ligands such as DGPC), it is believed that this binding interaction will be a major mechanism for its inhibition of MTP's lipid transporting function. However, since lomitapide can also bind to PDI and thus disrupt the binding interaction between PDI and MTPα, this effect of lomitapide may also partially contribute to its inhibition of the MTP function.

**Figure 5:**
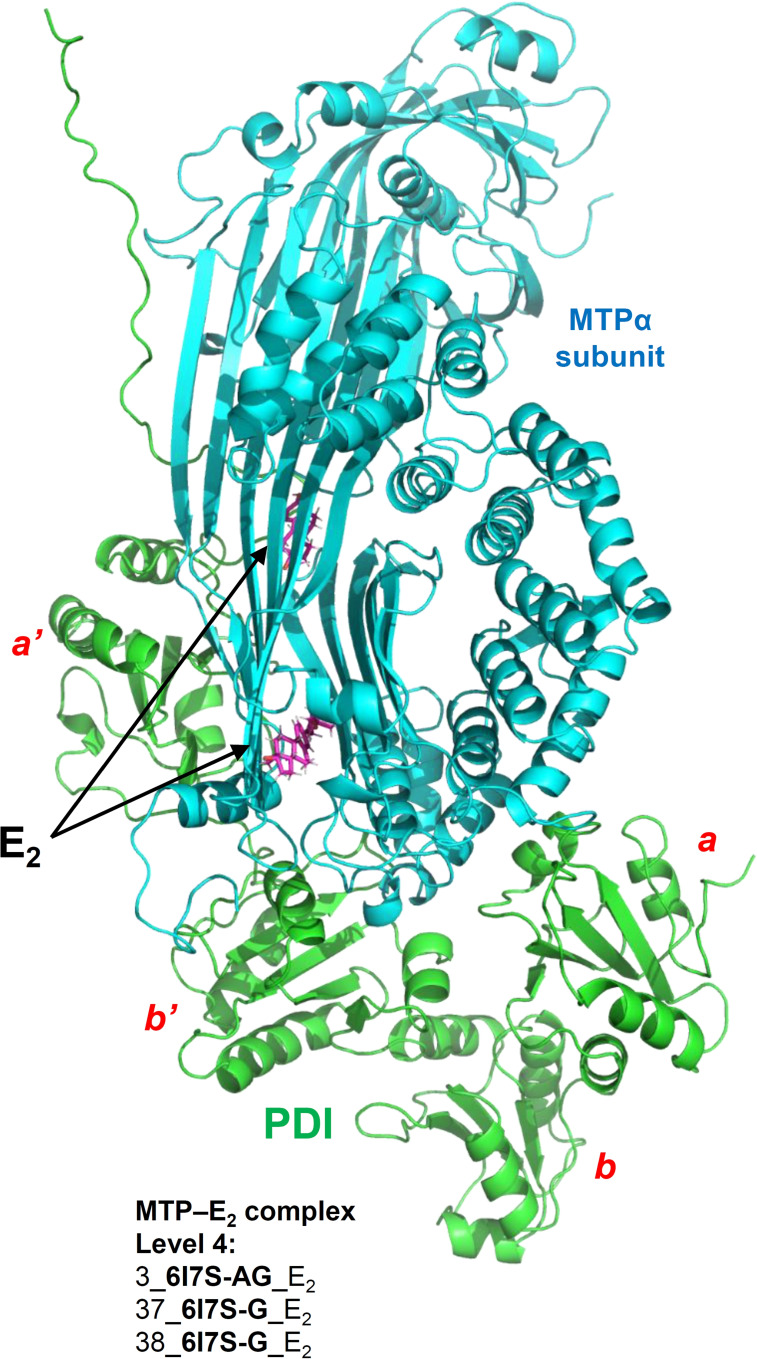
**The conformations of MTP–E_2_ complexes after production simulation (100 ns)**. Level 4: 3_**6I7S‐AG**_E_2_, 37_**6I7S‐G**_E_2_, 38_**6I7S‐G**_E_2_.

**Possible mechanism for MTP inhibition by E_2_ from both structure and energy perspectives**. According to the results from docking calculation and MD simulations, E_2_ can bind inside the lipid‐binding pocket in the MTP complex (Figure 5, level 4: 3_**6I7S‐AG**_E_2_, 37_**6I7S‐G**_E_2_ and 38_**6I7S‐G**_E_2_). Although the binding strength in the MTP–E_2_ complex (level 4: 3_**6I7S‐AG**_E_2_) is not as strong compared to the MTP–DGPC complexes (Figure 4B, level 1: 44_**6I7S‐G**_DGPC, 9_**6I7S‐AG**_DGPC), the large space in the lipid‐binding pocket can readily hold small ligands like E_2_. Based on the 9 shared amino acid residues in the MTP−E_2_ complex and MTP−DGPC complex (residues 643, 666, 674, 706, 707, 717, 765, 767 and 776; Table 3), it is very likely that the binding interaction of E_2_ at this lipid‐binding pocket will exert a competitive inhibition of the binding of phospholipids with MTP. However, due to the relatively lower binding strength of E_2_ at this binding site compared to lipid molecules, the degree of inhibition might be rather small.

**Figure 6:**
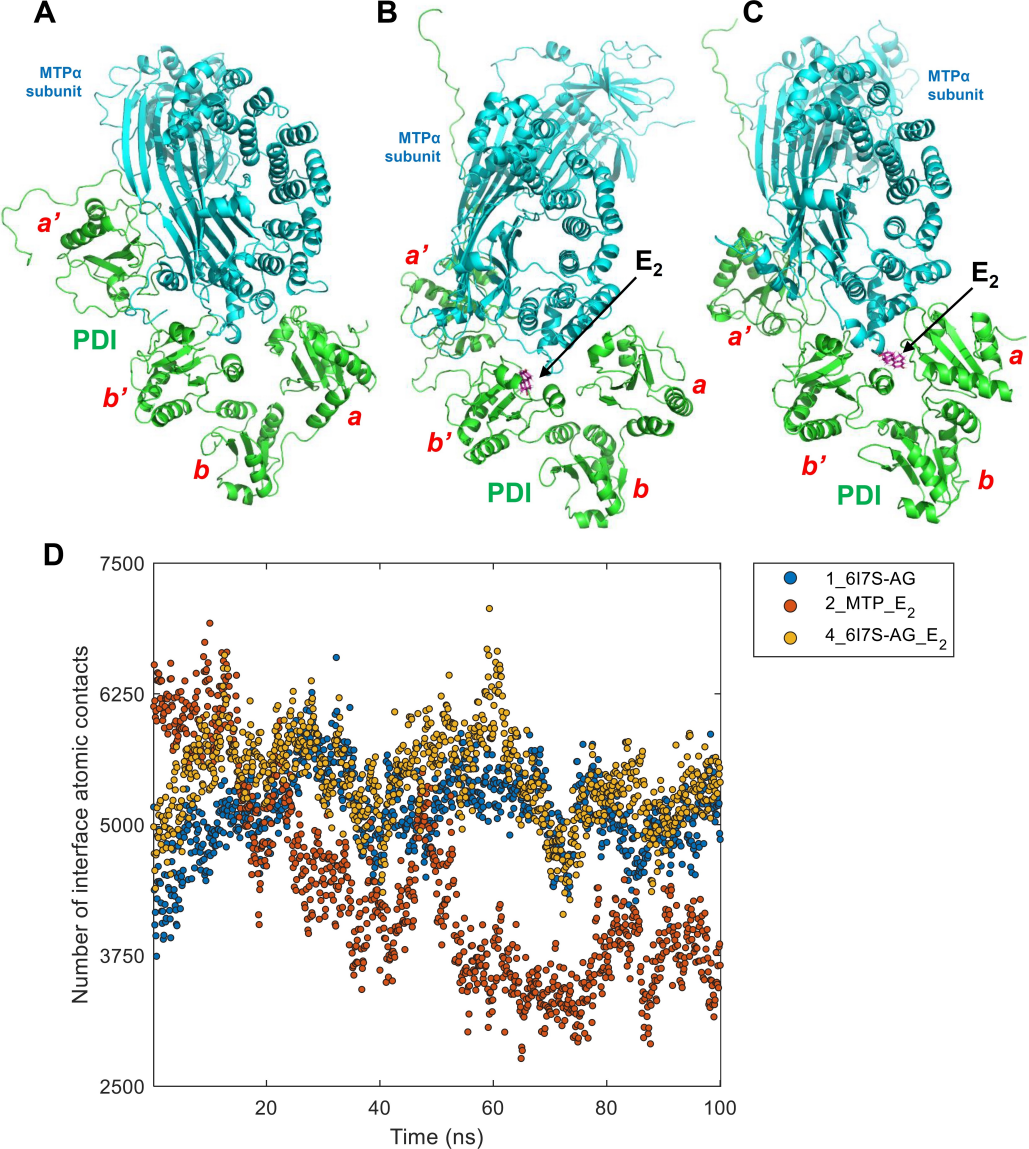
**The conformations of MTP complex and two MTP‐E_2_ complex with E_2_ binding at the interface between PDI and MTPα subunit after production simulation (100 ns), and the number of interface atomic contacts during the simulation processes. A**. MTP complex (1_**6I7S‐AG**). **B**. MTP–E_2_ complex with E_2_ binding on the *b’* domain (level 3: 2_**MTP**_E_2_). **C**. MTP–E_2_ complex with E_2_ binding in the space between the a and *b’* domains (level 4: 4_**6I7S‐AG**_E_2_). **D**. Number of interface atomic contacts between PDI and MTPα subunit in the three systems during the production simulation processes. The PDI, MTPα subunit and E_2_ are colored in green, cyan and purple, respectively.

In addition to its binding to the lipid‐binding pocket of MTP, we notice that E_2_ can also bind in the interface region between PDI and MTPα subunit (level 3: 2_**MTP**_E_2_ and level 4: 4_**6I7S‐AG**_E_2_) (Figure 6B and C). The two MTP–E_2_ structures (2_**MTP**_E_2_ and 4_**6I7S‐AG**_E_2_) are compared with the MTP complex without ligand (1_**6I7S‐AG**) (Figure 6A) using the number of interface atomic contacts between PDI and MTPα subunit, a metric reflecting the interface stability. As shown in Figure 6D, the numbers of interface atomic contacts in the systems 1 and 4 (1_**6I7S‐AG** and 4_**6I7S‐AG**_E_2_) are very similar during the production simulation processes. In system 4 (4_**6I7S‐AG**_E_2_), E_2_ binds in the space between ***a*** and ***b’*** domains, which do not influence the original interface atomic contacts in the MTP complex. However, the interface contact number in system 2 (2_**MTP**_E_2_) is lower than those in systems 1 and 4 (1_**6I7S‐AG** and 4_**6I7S‐AG**_E_2_). E_2_ binds to the ***b’*** domain of PDI in the MTP complex (2_**MTP**_E_2_), which disrupts the original interactions between PDI ***b’*** domain and MTPα subunit. If E_2_ binds to the ***b’*** domain of PDI first, and the PDI–E_2_ complex binds to MTPα subunit next, the interface stability would be reduced as shown in the previous work.^[17]^ If E_2_ binds in the space between the ***a*** and ***b’*** domains of PDI after the formation of the MTP complex, the interface stability does not appear to be affected.

In summary, the disruption of PDI–MTPα interaction resulting from the binding of E_2_ to PDI's ***b*** **’** domain likely is a major mechanism underlying MTP inhibition by E_2_. In addition, since E_2_ can also bind inside MTP's lipid‐binding pocket (with a moderate binding strength), this binding interaction may also partially contribute to its overall inhibition of MTP function through interrupting the MTP–phospholipid (DGPC) interaction.

**Open‐to‐closed or closed‐to‐open transitions of the PDI conformations**. In this study, we also characterize the conformational transitions of PDI in 34 PDI‐containing systems by using the following three metrics: **distance–0** between the ***a*** and ***a’*** domains, **angle–1** between the ***a***–***b***–***b’*** domains, and **angle–2** between the ***b***–***b’***–***a’*** domains. The domains are represented using the geometric centers of the backbone atoms. To differentiate the conformations, **distance–0**, **angle–1** and **angle–2** are evenly divided into 5 bins (named as bin‐1, bin‐2, bin‐3, bin‐4, bin‐5) based on the minimum and maximum values. The closed states are the conformations with at least two of the three metrics (**distance–0**, **angle–1**, **angle–2**) belonging to bin‐1 or bin‐2, and the open states are those with at least two of the three metrics (**distance–0**, **angle–1**, **angle–2**) belong to bin‐3 or bin‐4 or bin‐5. The other conformations are all considered as the in‐between states.

**Figure 7:**
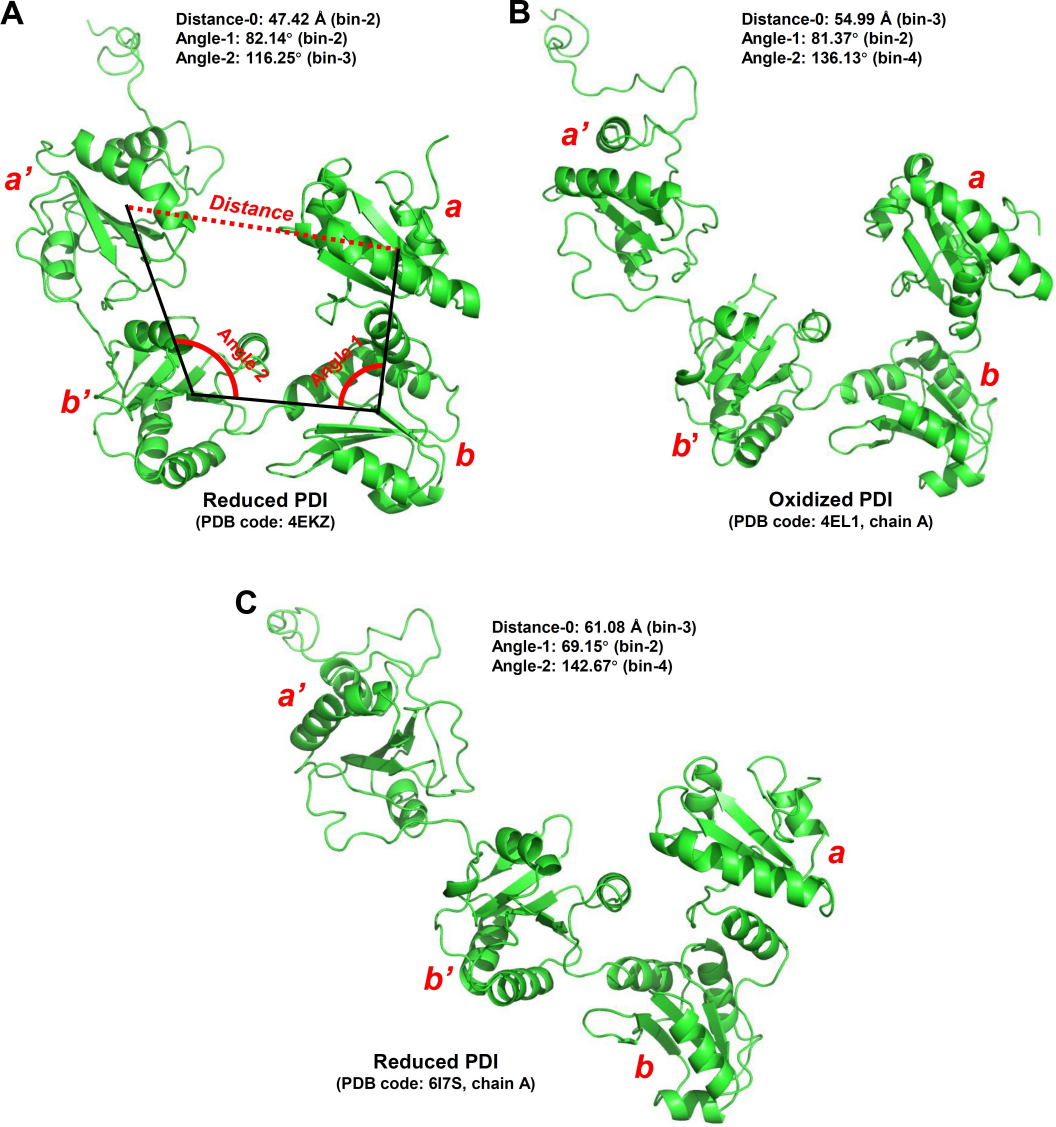
**Three metrics for evaluating the PDI conformational transitions in the intial PDI structures. A**. Reduced PDI (PDB code: **4EKZ**).^[15]^ **B**. Oxidized PDI (PDB code: **4EL1, chain A**).^[15]^ **C**. Reduced PDI (PDB code: **6I7S, chain A**).^[7]^ According to the mean values of the three metrics (**distance–0, angle–1 and angle–2**) during the equilibrium simulation process (2 ns), the reduced PDI (PDB code: **4EKZ**), oxidized PDI (PDB code: **4EL1, chain A**) and reduced PDI (PDB code: **6I7S, chain A**) are at open, closed and closed states, respectively.

The initial conformations of PDI and those with the minimum and maximum values of the three metrics are shown in Figure 5 and Supplementary Figure S5, respectively. According to the values of the three metrics, the initial reduced PDI conformation (PDI code: **4EKZ**)^[15]^ (Figure 7A) belongs to the closed state; both the initial oxidized PDI conformation (PDB code: **4EL1, chain A**)^[15]^ (Figure 7B) and the initial reduced PDI conformation extracted from the MTP complex (PDB code: **6I7S, chain A**)^[7]^ (Figure 7C) belong to the open states. The range of **distance–0** is between 31.63 Å and 89.68 Å (Supplementary Figures S5A and S5B), **angle–1** between 52.75° to 130.11° (Supplementary Figures S5C and S5D), and **angle–2** between 55.11° to 170.99° (Supplementary Figures S5E and S5F). The boundaries of **distance–0**, **angle–1** and **angle–2** are smaller than the respective minimum values in the experimental structures and also greater than the respective maximum values displayed in the experimental structures. PDI can readily adopt very closed confirmations as well as open conformations (Supplementary Figure S5). It is apparent that MD simulations provide more dynamic structural information than what was observed in earlier experimental studies.[^7^, ^15^]

**Figure 8:**
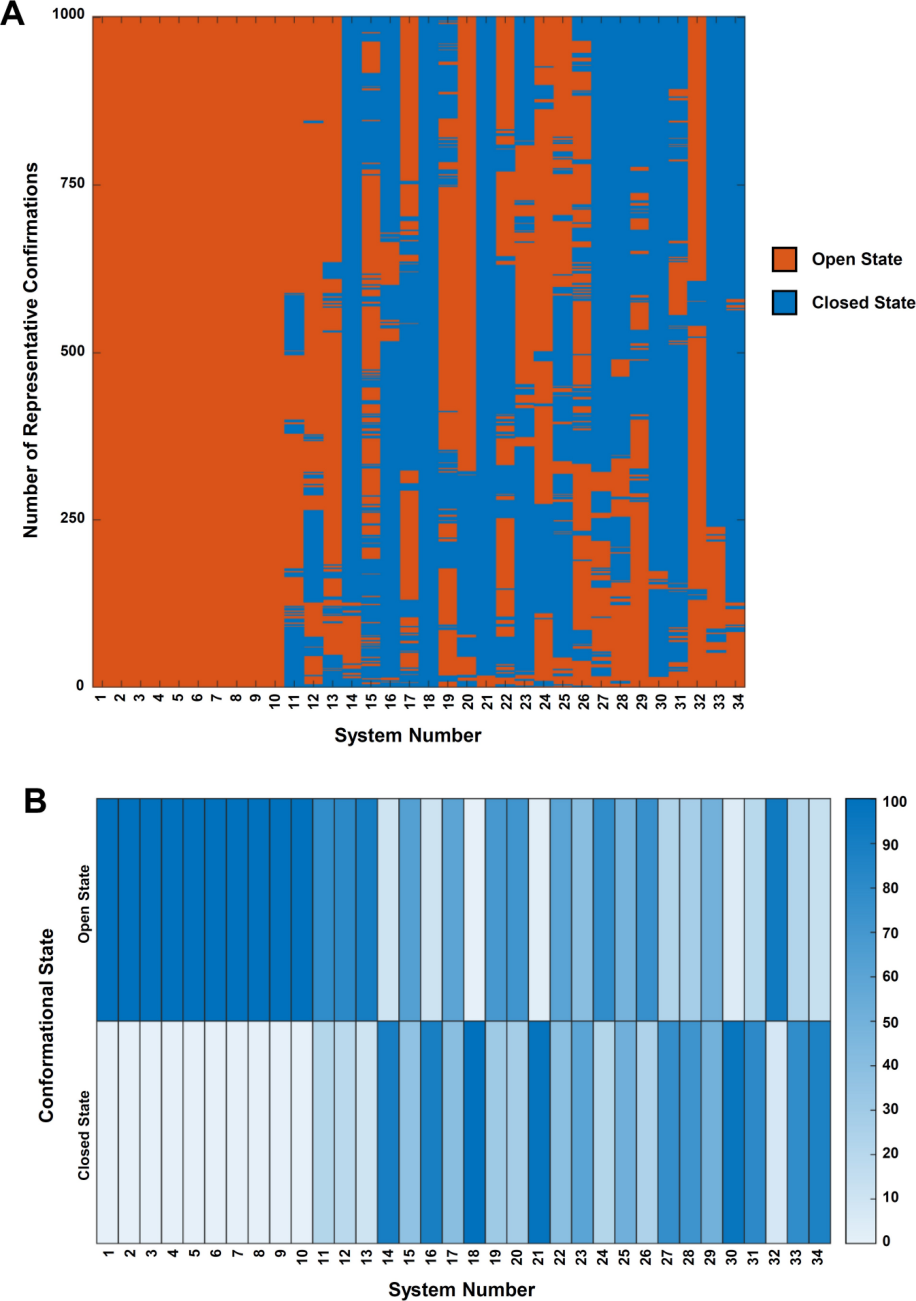
**The respresentative conformations of PDI in the open or closed state during the production simulation processes (100 ns) and the percentages of PDI's conformations at the open and closed states. A**. The represententative conformations of the open and closed states of PDI in 34 PDI‐containing systems. **B**. The percentages (%) of the open and closed states of PDI's conformations in 34 PDI‐containing systems.

**Figure 9:**
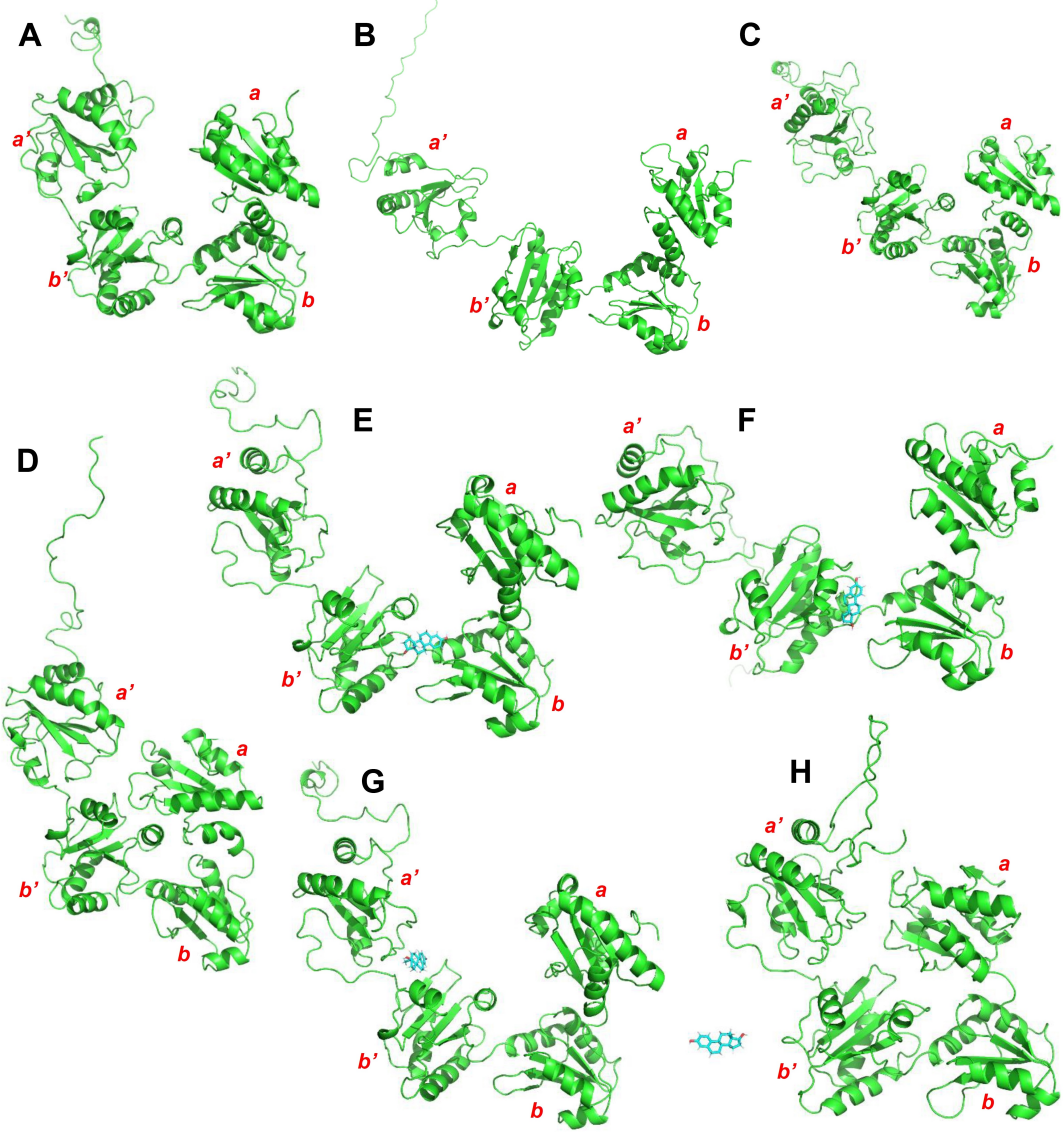
**Examples of PDI conformations for illustrating the open‐to‐closed and closed‐to‐open transitions during the production simulation processes (100 ns). A, B**. Closed‐to‐open conformational transition of the reduced PDI (11_**4EKZ**). The initial and final conformations of the reduced PDI are at closed and open states, respectively. **C, D**. Open‐to‐closed conformational transition of the reduced PDI (27_**6I7S‐A**). The initial and final conformations of the reduced PDI are at open and closed states, respectively. **E, F**. Open‐to‐open conformational transition of the oxidized PDI (20_**4EL1‐A_E_2_**). Both the initial and final conformations of the reduced PDI are at open states. **G, H**. Open‐to‐closed conformational transition of the oxidized PDI (21_**4EL1‐A_E_2_**). The initial and final conformations of the reduced PDI are at open and closed states, respectively.

The states of all representative PDI conformations during the production simulation process are shown in Figure 8A. Because of the presence of MTPα subunit, all the conformations of PDI in the MTP complex (systems 1 to 10) belong to the open states during the whole simulation processes. Nearly all PDI conformations in other systems except system 18 (18_**4EKZ**_DGPC) have both open and closed states during the processes. The percentages of open and closed states for the 34 systems are shown in Figure 8B (detailed data in Supplementary Table S7). The initial states of PDI (either reduced or oxidized) do not appear to affect the equilibrium (or conformational changes) between PDI's open and closed states. In addition, the binding of small molecule ligands to PDI also does not significantly affect the dynamic equilibrium process (or conformational changes) between PDI's open and closed states and their relative percentages. It is of note that this result is partially different from the results of a previous study.[^54^, ^55^, ^56^] Yang *et al*. investigated the PDI conformational changes using MD simulations and found that PDI adopts compact conformations in solvent.^[54]^ Okumura *et al*. and Chinnaraj *et al*. found that the oxidized PDI adopts open and closed conformations in dynamical equilibrium.[^55^, ^56^] However, the former observed that the confirmation of the reduced PDI is maintained at the closed states,^[55]^ and the latter observed that the reduced PDI predominantly exists in the open states.^[56]^ In the present study, both open‐to‐closed and closed‐to‐open transitions of PDI conformations can occur in the reduced and oxidized states in the simulation process. For instance, Figure 9A and B illustrate the closed‐to‐open conformational transition of the reduced PDI (11_**4EKZ**); Figure 9C and D illustrate the open‐to‐closed conformational transition of the reduced PDI (27_**6I7S‐A**); Figure 9E and F illustrate the open‐to‐open conformational transition of the oxidized PDI (20_**4EL1‐A**_E_2_); Figure 9G and H illustrate the open‐to‐closed conformational transition of the oxidized PDI (21_**4EL1‐A**_E_2_). These results on the apo PDI systems have been presented in the previous experimental or computational works.[^54^, ^55^, ^56^] Since the present work focuses on studying the binding of the selected ligands to PDI, additional parallel MD simulations are thus not performed on the apo‐PDI systems.

It is observed in this study that E_2_ dissociates from PDI during one of the MD simulation processes (Figure 9H). There are a few potential reasons for the dissociation: One is that the selected binding position and pose from the docking calculations are not the optimal ones. Another reason likely is the relatively weaker binding strength between the protein and ligand. Lastly, it should be noted that during real ligand–protein binding interactions, the binding or dissociation between a ligand and PDI is all a part of the dynamic equilibrium process. Even a ligand bound to a target protein (such as PDI) with very high affinity would still follow the dynamic equilibrium process; *i. e*., the ligand would not permanently stay bound to the protein. Hence, the observed dissociation in the simulation process likely is not an unusual phenomenon.

As a whole, the amplitudes of variation of **distance–0** and **angle–1** in the MTP complexes (systems 1 to 10) are smaller than those of PDI and PDI–ligand systems (systems 11 to 34) (Supplementary Figures S6A and S6B). The amplitudes of variation of **angle–2** in the MTP complexes (systems 1 to 10) are comparable to those of PDI and PDI–ligand complexes (Supplementary Figure S6 C). In the MTP complexes, the existence of MTPα subunit exerts restraints on **distance–0** (between ***a*** and ***a’*** domains) and **angle–1** (between ***a***–***b***–***b’*** domains), and the local movements between ***b***–***b’***–***a’*** domains (**angle–2**) are more dramatic than the local movements between ***a***–***b***–***b’*** domains (**angle–1**). The interactions between the ***a’*** domain and MTPα subunit are not strong enough to make the corresponding interfaces closely packing. Among the three interacting domains (***a***, ***b’***, ***a’***) with MTPα subunit, the ***b’*** domain may play a more important role in PDI–MTPα interaction. Combining the local movements with the global open‐to‐closed or closed‐to‐open transitions of PDI conformations, the binding process between PDI and MTPα subunit may be the mixture of conformation selection and induced‐fit model. In the PDI and PDI–ligand systems (system 11 to 34), the maximum MSFs (mean square fluctuations) of **distance–0**, **angle–1** and **angle–2** are 134.60 Å^2^, 245.83°^2^ and 512.22°^2^, respectively, and the minimum MSFs of **distance–0**, **angle–1** and **angle–2** are 9.53 Å^2^, 3.85°^2^ and 16.40°^2^, respectively. Among the systems with the highest MSFs, there exist reduced, oxidized and small ligand‐binding PDI (Supplementary Figures S6D, S6E, S6F). The percentages of conformations belonging to different bins (Supplementary Figures S6G, S6H, S6I) and the relative movements between different domains of PDI are not influenced by the reduced/oxidized states or by small ligand binding.

**Role of PDI in MTP function**. It is generally thought that PDI plays a multifunctional role in the formation of the MTP complex by catalyzing the oxidative folding of MTPα subunit and also by serving as molecular chaperone.[^4^, ^5^] We find that the conformations of MTPα subunit (35_**6I7S‐G**) are not changed during the production simulation (100 ns). Based on the simulation results, it appears that the molecular chaperone activity of PDI in the MTP complex may not be as important compared to its ability to catalyze the oxidative folding. In addition, PDI may also play a role in the interaction between MTP and apoB. Bradbury *et al*.^[57]^ reported that the residues 517–603 of MTPα subunit can interact with apoB at a site composed of residues 512–721. In the structure of the MTP complex,^[7]^ the residues 517–603 of MTPα subunit make interactions with PDI's ***a*** domain (Supplementary Figure S7). The disassociation between residues 517–603 of MTPα subunit and the ***a*** domain of PDI may be essential for the interactions between MTP and apoB. Wang *et al*. reported that PDI can recover the function of MTP in Ire1α‐deleted hepatocytes.^[58]^ It is expected that more insights regarding the necessity of PDI in MTP function may be gained from detailed study of the interactions between MTP and apoB.

## Conclusions

In this work, the binding interactions of three selected ligands (E_2_, lomitapide and a phospholipid molecule, DGPC) with PDI and with the MTP complex are investigated using computational modelling. The global conformational changes in PDI, *i. e*., open‐to‐closed transitions or closed‐to‐open transitions, appear to be not affected by the ligand binding and by the interconversion between its reduced and oxidized state. It is found that lomitapide and DGPC can bind inside the lipid‐binding pocket inside the MTP complex with high stabilities. E_2_ can bind in the interface region between PDI and MTPα subunit, although it can also inside the lipid‐binding pocket. In addition, both lomitapide and E_2_ can bind to PDI's ***b’*** domain. The residues 643, 666, 671, 706, 707, 717, 765, 767 and 776 in the lipid‐binding pocket of MTPα subunit are important for the binding interactions of E_2_, lomitapide and DGPC, and the residues 240, 249, 256, 258, 301, 304 and 318 of PDI's ***b’*** domain are involved in its binding interaction with E_2_ and lomitapide. These residues are believed to be important structural components in designing inhibitors for PDI and MTP complex. This work provides mechanistic insights at the amino acid levels concerning the pharmacological actions of lomitapide, the estrogen receptor‐independent lipid‐modulating effect of E_2_, and the interactions between DGPC and the MTP complex. In addition, the protocols employed in this work might also be of practical utility in the analysis of protein–ligand interactions in other cases.

## Author Contributions

Conceptualization, Y.X.Y.; B.T.Z.; methodology, Y.X.Y.; formal analysis, Y.X.Y., B.T.Z.; investigation, Y.X.Y., P.L.; B.T.Z.; resources, B.T.Z.; data curation, Y.X.Y.; P.L.; writing – original draft preparation, Y.X.Y., B.T.Z.; writing – review and editing, Y.X.Y., B.T.Z.; visualization, Y.X.Y.; supervision, B.T.Z.; project administration, B.T.Z.; funding acquisition, B.T.Z. All authors have read and agreed to the published version of the manuscript.

## Conflict of Interests

All the authors have read the manuscript and declared no conflict of interest.

1

## Data Availability

The data that support the findings of this study are available from the corresponding author upon reasonable request.
